# A pilot study for channel catfish whole genome sequencing and *de novo *assembly

**DOI:** 10.1186/1471-2164-12-629

**Published:** 2011-12-22

**Authors:** Yanliang Jiang, Jianguo Lu, Eric Peatman, Huseyin Kucuktas, Shikai Liu, Shaolin Wang, Fanyue Sun, Zhanjiang Liu

**Affiliations:** 1 The Fish Molecular Genetics and Biotechnology Laboratory, Department of Fisheries and Allied Aquacultures, Program of Cell and Molecular Biosciences, Aquatic Genomics Unit, 203 Swingle Hall, Auburn University, Auburn, AL 36849, USA; 2 Department of Psychiatry and Neurobiology Science, University of Virginia, 1670 Discovery Dr. Suite 110, Charlottesville, VA 22911, USA

## Abstract

**Background:**

Recent advances in next-generation sequencing technologies have drastically increased throughput and significantly reduced sequencing costs. However, the average read lengths in next-generation sequencing technologies are short as compared with that of traditional Sanger sequencing. The short sequence reads pose great challenges for *de novo *sequence assembly. As a pilot project for whole genome sequencing of the catfish genome, here we attempt to determine the proper sequence coverage, the proper software for assembly, and various parameters used for the assembly of a BAC physical map contig spanning approximately a million of base pairs.

**Results:**

A combination of low sequence coverage of 454 and Illumina sequencing appeared to provide effective assembly as reflected by a high N50 value. Using 454 sequencing alone, a sequencing depth of 18 X was sufficient to obtain the good quality assembly, whereas a 70 X Illumina appeared to be sufficient for a good quality assembly. Additional sequencing coverage after 18 X of 454 or after 70 X of Illumina sequencing does not provide significant improvement of the assembly. Considering the cost of sequencing, a 2 X 454 sequencing, when coupled to 70 X Illumina sequencing, provided an assembly of reasonably good quality. With several software tested, Newbler with a seed length of 16 and ABySS with a K-value of 60 appear to be appropriate for the assembly of 454 reads alone and Illumina paired-end reads alone, respectively. Using both 454 and Illumina paired-end reads, a hybrid assembly strategy using Newbler for initial 454 sequence assembly, Velvet for initial Illumina sequence assembly, followed by a second step assembly using MIRA provided the best assembly of the physical map contig, resulting in 193 contigs with a N50 value of 13,123 bp.

**Conclusions:**

A hybrid sequencing strategy using low sequencing depth of 454 and high sequencing depth of Illumina provided the good quality assembly with high N50 value and relatively low cost. A combination of Newbler, Velvet, and MIRA can be used to assemble the 454 sequence reads and the Illumina reads effectively. The assembled sequence can serve as a resource for comparative genome analysis. Additional long reads using the third generation sequencing platforms are needed to sequence through repetitive genome regions that should further enhance the sequence assembly.

## Background

Channel catfish, *Ictalurus punctatus*, is the major aquaculture species in the United States, accounting for over 60% of all U.S. aquaculture production. Channel catfish is regarded as one of the best characterized species serving as a model for teleost immune studies [1], and an important model species for study of toxicology and reproductive physiology [2]. The channel catfish genome is estimated to be 1 Gb in size http://www.genomesize.com and is highly AT-rich, with 60.7% A+T [3]. The catfish genome contains one main type of tandem repeats named as Xba elements [4] and several types of dispersed repetitive elements with the mariner/Tc1 DNA transposons as the leading type of dispersed repetitive elements (4-5% of the genome), followed by retrotransposons (3-4% of the genome), Mermaid, Merman, and other SINE elements (~1.5% of the genome), LINES (~1.5% of the genome) and various types of short sequence repeats such as microsatellites (~3% of the genome) [3,5-8].

At present, a number of genomic tools and resources have been developed in catfish, including bacterial artificial chromosome (BAC) libraries [9,10], BAC-based physical maps [11,12], genetic linkage maps [13-15], a large number of ESTs [2,16], over 1700 unique full length cDNAs [17], over 60,000 BAC end sequences [3,7], and a large number of identified molecular markers such as microsatellites and single nucleotide polymorphisms [2,18]. Whole genome sequencing of catfish is underway, and this project was conducted as a pilot study to define the parameters important for the generation of the whole genome sequence assembly.

A major limitation of eukaryotic genome sequencing is the costs involved in sequencing. In recent years, however, advances in sequencing technologies have allowed drastic reduction in sequencing costs. Among many sequencing platforms, the second generation of sequencing technologies such as 454 sequencing, Illumina sequencing, and SOLiD sequencing are the most commonly used sequencing platforms. A common feature of these sequencers is their relatively short sequencing reads, making subsequent sequence assembly a great challenge. Such challenges become even more significant when dealing with large and complex eukaryotic genomes. Teleost genome, known to have gone through a third round of whole genome duplication [19], poses additional challenge when coupled with the short sequencing reads. In consideration of such complexities, Quinn et al. [20] conducted a pilot study with eight pooled BAC clones covering approximately 1 Mb of the Atlantic salmon genome with 454 GS FLX pyrosequencing, and concluded that it was difficult to achieve good levels of genome sequence assembly with 454 sequencing with the tetraploid genome. However, with the diploid European sea bass, Kuhl et al. [21] was able to generate large superscaffolds (13.2-17.5 Mb) with 17-39X coverage of pooled BACs using pyrosequencing. Apparently, genome complexity as well as existing genome resources can influence the outcomes of assembly of sequences generated from next generation sequencing. In this study, based on the existing catfish BAC-based physical map, 24 pooled BAC clones covering around 1 Mb catfish genome were sequenced by using both pyrosequencing and Illumina sequencing technologies. In addition with the existing BAC end sequences, we aim to take full advantages of multiple sequencing technologies and existing genetic resources for the upcoming catfish whole genome sequencing.

Several *de novo *assembly software packages have been developed for assembling genome sequences generated from the next-generation sequencing platforms. Two basic graph based algorithms exist for assemblers: The first one is based on overlap-layout-consensus graphs, such as Newbler [22] and MIRA [23]. In these packages, the algorithm computes all pair-wise overlaps between reads to build an overlap graph. Then, the overlap graph is used to compute a layout of reads and consensus sequence of contigs. The second algorithm is based on de-Bruijn graphs, such as the algorithm used in Velvet [24], ABySS [25], and several other packages in which sequence reads are broken into smaller sequences of DNA, referred to as K-mers, where K denotes the length of these smaller sequences [26]. The de-Bruijn graph is built based on the overlaps of length K-1 between these K-mers rather than the actual reads. The assembly algorithms and their implementations become typically complex with large volumes of sequence data.

The objectives of this pilot study was to determine the framework for proper levels of genome coverage using Illumina sequencing, 454 sequencing, and a combination of 454 and Illumina sequencing when both technical factors and economic factors were considered, to compare the assembly of sequences using different software packages with various parameters, and to develop a cost-effective *de novo *assembly strategy for the upcoming catfish whole genome sequencing. Here we report sequencing and assembly of twenty-four BAC clones of the largest contig of catfish physical map using 454 sequencing, Illumina sequencing, and a combination of these sequencing technologies, and compare their assembly using several *de novo *assembly softwares. Based on the assembly, we attempted to identify conserved syntenies among catfish and several fish species whose genome sequences are available using BLAST [27] sequence similarity comparisons.

## Results and discussion

### Generation of short sequencing reads from pooled catfish BAC clones

To assess the *de novo *assembly strategy for the catfish whole genome sequencing project, twenty-four BAC clones were selected from the largest contig (contig0241) of the BAC-based catfish physical map. Individual clones included in the contig were pooled together and sequenced by using both 454 and Illumina sequencing technologies. Based on the physical map, the estimated length of the contig spanned approximately 1 Mb long. As shown in Table 1, a total of 200,901 from 454 single-end reads, 29,067,404 from Illumina 72-bp paired-end reads from a 350-bp insert library were generated. After adaptor and BAC vector trimming, and low quality reads filtering, a total of 41 Mb 454 sequences and 1,245 Mb Illumina paired-end sequences were obtained (Table 1). These represented approximately 40 X and 1,200 X sequence coverage of the contig for 454 and Illumina sequencing, respectively. All the sequences have been deposited to the Catfish Genome Database cBARBEL [[28], http://www.animalgenome.org/catfish/cbarbel/contig0241].

**Table 1:** Sequencing statistics using 454 and Illumina sequencing platforms.

Primary sequence data	454	Illumina
Total number of reads	200,901	29,067,404
Total number of reads after trimmed	141,227	20,829,734
Average trimmed read-length (bp)	292	60
Total accumulative length of all trimmed reads (Mb)	41	1,245

### Comparison of *de novo *assemblers for 454 reads and Illumina reads

A number of sequence assemblers have been developed recently to cope with sequences generated from the next generation sequencers. To determine the most appropriate assembler and its associated K-values (as appropriate) to use for the *de novo *assembly of 454 reads and Illumina reads, we compared several existing assemblers: Newbler, Velvet, ABySS, MIRA and a commercial software, CLC Genomics Workbench http://www.clcbio.com. The Celera Assembler V6.1 was also considered, but our Illumina sequences generated a while ago was not long enough for the assembler. The metrics used to evaluate the assembly included N50, the average contig size, the maximum contig size and the number of contigs.

As highlighted in Table 2, for 454 sequences, Newbler allowed the most effective assembly, compared to Velvet, MIRA, or CLC Genomics Workbench. With Newbler, the best assembly was achieved at seed length (*i.e*. "K" value of the K-mer matching) of 16 at which 282 of contigs were assembled, with N50 of 8,674 bp, average contig size of 3,486 bp, and maximum contig length of 28,736 bp. For Illumina sequences, ABySS and Velvet provided more effective assemblies than CLC Genomics Workbench (Table 2). The best assembly using ABySS was achieved at K-value of 60 at which 386 contigs were assembled with N50 of 6,870, average contig size of 2,496 bp, and maximum contig size of 22,594 bp. Velvet at K-value of 29 generated a similar assembly to that of ABySS with N50 of 6,319 bp, average contig size of 1,703 bp, and maximum contig size of 35,339 bp, but with significantly greater number of contigs. Similar results were found in a recent study using ABySS [29].

**Table 2:** Comparison of assembly output with 454 reads and Illumina reads using different assemblers with different K-values.

	Assembler	K-value/Seed length	N50 (bp)	Maximum contig size (bp)	Average contig size (bp)	Number of contig
**454**		10	8,497	28,747	3,387	290
		11	7,524	28,723	3,457	284
		12	8,164	26,457	3,520	279
	**Newbler**	13	8,670	31,603	3,478	283
		14	8,635	28,741	3,523	279
		15	8,164	35,657	3,487	282
		**16**	**8,674**	**28,736**	**3,486**	**282**
	
		25	653	4,678	437	1,787
		35	736	3,717	468	1,645
		45	744	3,958	505	1,409
	**Velvet**	55	731	3,515	513	1,213
		65	718	2,730	556	943
		75	691	3,060	574	757
		85	621	3,538	584	622
		95	595	3,291	600	488
	
	**MIRA**		1,331	8,705	856	1,254
	
	**CLC Genomics Workbench**		1,239	7,621	988	1,220

**Illumina**		21	4,196	23,500	1,242	954
		25	5,678	40,818	1,495	809
		**29**	**6,319**	**35,339**	**1,703**	**705**
		33	4,745	32,597	1,744	659
		37	4,507	22,638	1,725	637
	**Velvet**	41	4,355	15,944	1,712	602
		45	3,619	19,463	1,677	579
		49	3,605	18,297	1,849	511
		53	3,381	18,243	1,813	518
		57	3,025	15,219	1,631	552
		61	3,388	15,148	2,011	390
	
		30	2,296	13,010	1,027	1,013
		35	2,493	16,400	1,132	933
		40	3,647	20,868	1,439	716
	**ABySS**	45	4,463	20,727	1,681	578
		50	5,481	22,931	2,084	453
		55	5,459	23,830	2,316	401
		**60**	**6,870**	**22,594**	**2,496**	**386**
		64	4,285	16,790	1,722	524
	
	**CLC Genomics Workbench**		823	8,964	454	3,572

### Assembly strategy for the combination of 454 and Illumina reads

The hybrid assembly is attempted by combining all reads from both 454 and Illumina platform. Using the combination of two types of sequencing reads, we tested Velvet, ABySS, Newbler and CLC for hybrid assembly. Using a single assembler, ABySS with K-value 60 provided a good quality assembly, with N50 value of approximately 9.1 Kb and a maximum contig size of approximately 26 Kb. Newbler v2.6 performed better for the assembly of the same sequence data that generated a N50 value of approximately 12.5 Kb and a maximum contig size of 39 Kb. However, assembly derived from 454 reads plus Illumina reads by using a single assembler did not provide much advantage as compared with the assembly of using only 454 reads. Rather than using a single assembler for the hybrid assembly, it is crucial to develop a suitable strategy by using several assemblers for the assembly of combination of different types of sequencing reads.

**Figure 1:**
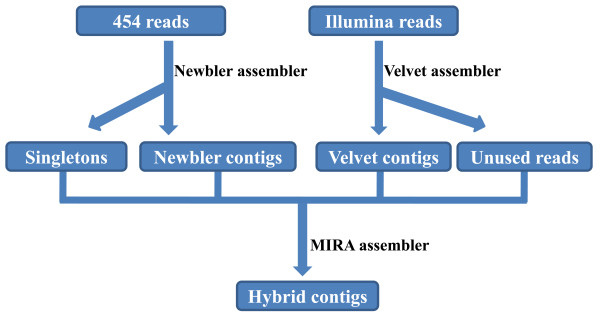
**Hybrid assembly strategy pipeline**. All 454 reads were assembled by using Newbler assembler with seed length 16, minimum overlap length 40 and minimum overlap identity 95%. All Illumina reads were assembled by using Velvet assembler with K-value 29. MIRA assembler was then used to generate the hybrid contigs with minimum overlap 40.

The strategy for the hybrid assembly is shown in Figure 1. Initially, 454 sequences were assembled using Newbler and Illumina sequences were assembled using Velvet. Velvet rather than AbySS was used for the assembly of Illumina reads because some of the contigs generated by ABySS contained internal gaps since ABySS has the capability to merge contigs using paired-end information, and these internal gaps may influence the subsequent assembly attempts. After the initial separate assembly, the contigs assembled and the remaining unassembled sequences were subjected to a secondary assembly using MIRA. This additional assembly step with MIRA maximized the complementary advantages of 454 and Illumina reads, resulting in the assembly of 193 contigs. The maximum contig size was 71,434 bp. All 75 BAC end sequences from the same physical map contig generated in our previous studies [3,7] were aligned to the hybrid assembled contigs, suggesting that the sequence coverage is near completion and gaps are small, as expected from the size estimation. The contigs sequences have been deposited to Catfish Genome Database cBARBEL http://www.animalgenome.org/catfish/cbarbel/contig0241.

Several studies have utilized two-step assembly strategy for the assembly of Illumina and 454 sequences: In an effort to sequence the whole genome of a model fungi *Sordaria macrospora*, Nowrousian et al. [30] pre-assembled 454 reads first, and then constructed an assembly by using the combined raw data from both Illumina and 454 reads using Velvet assembler; In a different study, the whole genome sequence reads from a bacteria, *Geobacter sulfurreducens*, Illumina reads were alone pre-assembled first and the contigs generated from this assembly plus the singletons and the 454 reads were then assembled using Newbler [31].

### Depth of sequence coverage required for effective *de novo *assembly

**Figure 2:**
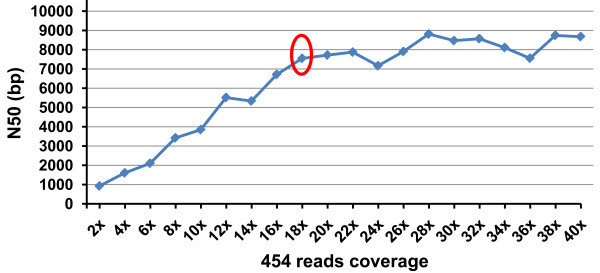
**Comparison of N50 values assembled using Newbler with various sequencing depth of 454 sequence reads**. Red circle indicates the sequence depth at which additional sequencing started to loss power for effective sequence assembly.

**Figure 3:**
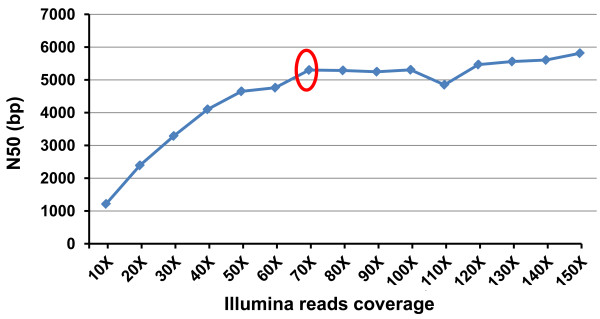
**Comparison of N50 values assembled using ABySS with various sequencing depth of Illumina sequence reads**. Red circle indicates the sequence depth at which additional sequencing started to loss power for effective sequence assembly.

The depth of sequence coverage needed for effective sequence assembly depends on the genome size, the GC content [32] and repetitive elements content of the organism, and the sequence read lengths. In addition, error correction algorithms used in assemblers can be utilized effectively when the depth of coverage is high [33]. The Beijing Genomics Institute sequenced the Giant Panda genome using 75 bp Illumina reads at a coverage of 50 X [34] leaving a small number of gaps that may be filled by Sanger sequencing [33]. In this pilot project, we evaluated the influence of depth of sequence coverage of different types of sequencing data sets on the overall assembly. As shown in Figure 2, the N50 values increased almost linearly until the 454 sequences reached 18 X coverage. After 18 X sequence coverage, additional 454 sequencing generated slightly greater N50 values, but was not cost effective any longer. We understand that our 454 sequences were generated with older chemistry and overall sequence length was relatively short, but the point is that with relatively short 454 sequences, assembly quality is initially positively correlated with sequence coverage. However, when the sequence coverage reached a certain level (here 18 X), additional sequence coverage was no longer effective (Figure 2). Similarly, with Illumina sequencing, the N50 values increased almost linearly with the sequence coverage up to 70 X sequence coverage (Figure 3). However, additional Illumina sequencing was ineffective in providing a better sequence assembly, as reflected in the plateau N50 values after 70 X sequence coverage (Figure 3). Apparently, the assembly that can be achieved may be related to sequence read lengths, e.g., the higher level of genome coverage may not help if the sequence read length is short.

**Figure 4:**
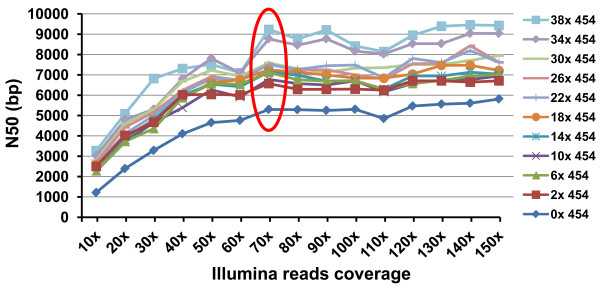
**Comparison of N50 values assembled with various sequencing depth combination of 454 and Illumina reads**. The assemblies were achieved by using the two step approach (Newbler for 454 data, Velvet for Illumina data for initial assembly followed by assembly with MIRA, for details, see materials and methods), with various sequencing depth combination of 454 sequence reads and Illumina sequence reads. Red circle indicates the sequence depths at which additional sequencing started to loss power for effective sequence assembly.

An effective sequencing strategy is to use a hybrid sequencing strategy of Illumina plus 454 sequencing. In order to determine if a hybrid approach is cost effective, we evaluated assembly of the contig using various 454 and Illumina sequence coverage combinations. As shown in Figure 4, inclusion of 454 sequencing very effectively increased N50 of the assemblies. Interestingly, a small sequence coverage of 454 sequencing provided almost as effective sequence assembly as a large 454 sequence coverage. Only when the 454 sequences reached a very high sequence coverage (over 30 X coverage), the overall sequence assembly was more effective with much greater N50. N50 values of 454 sequences at 6-30 X provided similar assemblies. Similar but different assembly patterns were observed with various sequence coverages of Illumina sequences: with or without 454 sequences, the N50 values of the assemblies increased almost proportional to Illumina sequence coverages up to 70 X sequence coverage; thereafter, additional Illumina sequences were not effective in increasing the N50 values. Considering the cost for the two sequencing platforms, 454 sequencing is much more expensive than Illumina sequencing, e.g., 2 X catfish whole genome sequencing with 454 sequencing technology will cost $25,000, which is almost the cost to get 140 X catfish whole genome sequences using Illumina sequencing. Taking the cost into consideration, it appears that the assembly of 2 × 454 sequences and 70 X Illumina sequences was the most effective (Figure 4). The importance of longer reads should be noted. In the catfish genome, and perhaps many fish genomes as well, there are long repetitive elements such as the mariner-like DNA transposons. Long reads helps to get through such long repetitive regions. Working with the genome of *Rhodopseudomonas palustris*, Schadt et al. [35] demonstrated that long reads generated from PacBio sequencing can help resolve assembly difficulties in a repetitive region spanning 1.5 kb, which otherwise cannot be assembled by using Illumina sequencing. In our case with the catfish genome segment here, the failure of contig assembly was mostly caused by repetitive sequences, and therefore, the adoption of PacBio, or similar long sequencing platforms, should enhance the contig assembly.

### Comparison of assembly with Illumina paired-end and single-end reads and scaffolding

Paired-end reads are the reads in pairs such that each pair is of a known distance from each other. Although the cost associated with library construction of single-end reads is much cheaper than that of paired-end reads, because of their linked nature, paired-end reads are important for *de novo *assembly because they can improve the assembly by spanning repeat regions, anchoring reads to the sequence within repeat regions, and ordering of contigs into larger scaffolds [32]. To investigate the advantage of paired-end reads over single-end reads, we compared the assembly between Illumina paired-end and single-end reads with the same set of sequence data using ABySS with a K-value of 60. Table 3 clearly shows the advantage of paired-end reads over single-end reads for *de novo *assembly. The N50 value with Illumina paired-end reads is approximately 3.6 Kb, which is 5.6 times larger than the N50 value of single-end reads assembly (652 bp). Similarly, the maximum contig size with Illumina paired-end reads is almost 21 Kb, while the maximum contig size of single-ends reads assembly was only 3.5 Kb (Table 3). The difference in the number of assembled contigs is more dramatic, with 716 contigs from paired-end reads assembly but 2,230 contigs with single-end reads assembly. In a recent study, the giant panda whole genome draft was completed by using multiple paired-end reads libraries with different insert size [34], which initiated the eukaryotic genome sequencing and assembly based mostly on the next generation sequencing platform. Clearly, use of multiple libraries in various sizes would increase the effectiveness of sequence assemblies, but that was not tested in this study.

**Table 3:** Comparison of assemblies from Illumina paired-end reads (PE) and single-end reads (SE).

	N50 (bp)	Maximum contig size (bp)	Average contig size (bp)	Number of contig
**PE**	3,647	20,868	1,439	716
**SE**	652	3,530	442	2,230

The importance of paired reads is not limited to contig assembly. They are even more important in scaffolding by bringing separate contigs together into larger scaffolds. For instance, when the paired-end reads of Illumina sequences were used, the 193 contigs were brought together into 68 scaffolds. The scaffolding capacity of paired-end reads is under estimated in this study because we used a library with small insert size of 350 bp. The scaffolding capacity of paired-end reads should be much greater if larger insert libraries were used. The BAC end sequences helped in scaffolding, but were less effective than expected because of the small number of the available mate paired BAC end sequences within this contig. Of the 75 BAC end sequences falling within this contig, only 29 were mate paired reads. Along with the paired-end reads of Illumina sequences, these BAC end sequences allowed assembly of 193 contigs into 61 scaffolds with the largest scaffold of 684,936 bp.

In order to have a sense of comparison of the sequence assembly in this pilot study with similar studies or those achieved through whole genome sequencing, we summarized contig size information in Table 4. In terms of N50 and average contig sizes, the assemblies presented here with catfish is similar to those of the pilot study with Atlantic salmon [20]. A direct comparison of the assemblies here with only one million base pairs and those with the whole genome sequences is difficult because the sequence complexities and sequencing strategies were different. However, if the similar results from this pilot study could be extended to the genome scale with similar genome coverage, the results to be achieved with catfish whole genome sequencing would be similar to those achieved with *Tetraodon*, Fugu, or Atlantic cod.

**Table 4:** Comparison of assembly statistics of fully sequenced fish genomes^1 ^and some pilot studies^2^.

Species	Contig/scaffold^3 ^N50(bp)	Number of contigs	Maximum contig/scaffold^3 ^size (bp)	Average contig size (bp)	Total bases
**Zebrafish^1^**	25,000	119,136	N/A	11,856	~1 Gb
**Medaka^1^**	9,800	36,494	N/A	19,181	~700 Mb
**Tetraodon^1^**	16,000	49,609	258,000	6,289	~312 Mb
**Atlantic cod^1^**	7,128	135,024	117,463	4,658	~629 Mb
**Fugu^1^**	858,115**^3^**	45,024	7,245,445**^3^**	8,729	~393 Mb
**Atlantic salmon^2^**	13,455	158	38,211	6,827	~1 Mb
**Catfish^2^**	13,123	193	71,434	5,313	~1 Mb

### Repetitive content analysis and annotation of the assembled region

Repetitive content of the assembly were assessed by using RepeatMasker http://www.repeatmasker.org against zebrafish repeat database. A total of 140,019 bp (13.6%) sequences were masked. The content of various types of repetitive elements in the assembled region is presented in Table 5. The most abundant type of repeat was found to be DNA transposons totaling 8.38% of the assembly, which confirmed our previous findings that DNA transposons were the most abundant type of repetitive elements in the catfish genome [3,6,7]. However, the 8.38% of DNA transposons found here was almost twice the amount as previously assessed [6].

**Table 5:** Summary of repetitive elements in the assembled region of catfish genome.

	**Transposable elements**				**Tandom repeats**			**Total**
			
	**DNA transposons**	**Retroelements**			**Satellites**	**Simple repeats**	**Low complexity repeats**	
						
		**SINEs**	**LINEs**	**LTRs**				
		
**Length (bp)**	86,175	6,527	9,040	12,485	1,193	17,223	7,955	
**% of sequences**	8.38%	0.63%	0.88%	1.21%	0.12%	1.67%	0.77%	13.66%

Overall, 98.5% of all reads were assembled. That is to say, 1.5% of the generated sequences were not assembled. Apparently, many unassembled sequences were very short reads, but they are probably also repetitive in nature so that prohibit themselves to be assembled. BLAST was used to assess the nature of the unassembled sequences against the *nr *database with a cutoff E-value of 1e-5. Most hits were repetitive elements such as transposable elements (~18% of unassembled reads), retrotransposons reverse transcriptase-like sequences (~8%), zinc-finger protein-like (~1%), recombinase-like protein (~1%), among others. In addition, the unassembled sequences were also used for BLAST search against themselves to determine if they are highly repetitive sequences themselves. Approximate 79% of the unassembled reads can hit more than 10 other reads, 55% of which hit more than 100 other reads. Taken together, these results indicated that the unassembled reads are located in the repetitive region of the genome, resulting in gaps when conducting *de novo *assembly. Therefore, long reads are crucially important to go through such repetitive regions of the genome.

**Figure 5:**
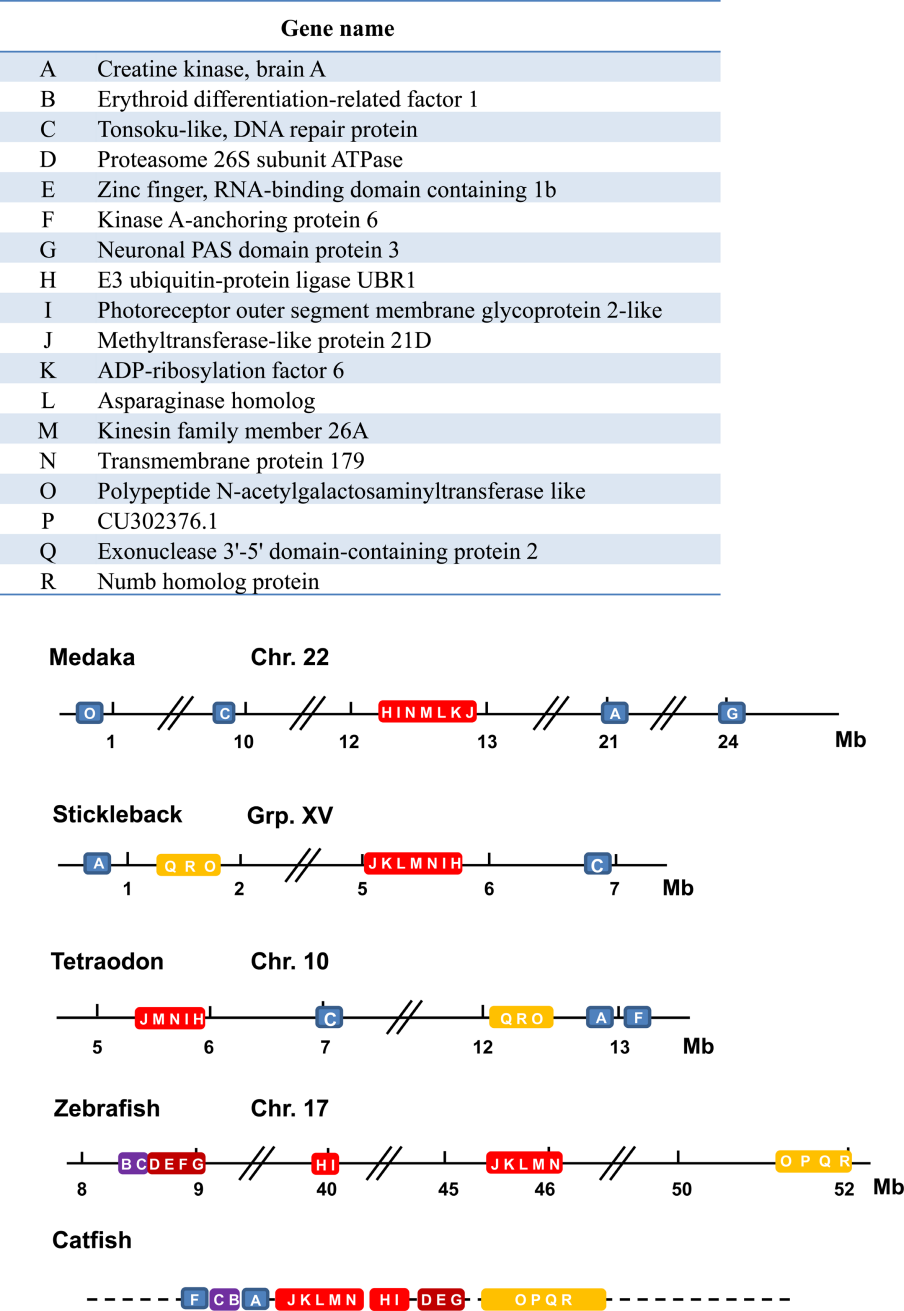
**Schematic presentation of conserved syntenic regions among catfish, zebrafish, medaka, stickleback, and *Tetraodon***. A list of the 18 identified genes in the sequenced region is shown on the upper panel of the figure, with each gene abbreviated using a letter to make it easier to present the conserved syntenies at the lower panel of the figure. For instance, the letter A represents creatine kinase (brain type A). Figure was drawn not proportionally to the scale as indicated by the double slashes. Same color indicates conserved syntenic blocks.

The repeat masked contig sequences were searched against NCBI *nr *database using both BLASTX searches and GENSCAN gene model prediction algorithm [36] to identify the genes in the assembled region. A total of 18 genes were identified (Figure 5). These genes included creatine kinase (brain A type), erythroid differentiation-related factor 1, tonsoku-like DNA repair protein, proteasome 26 S subunit ATPase, zinc finger RNA-binding domain containing protein 1 b, kinase A (PRKA)-anchor protein 6, neuronal PAS domain protein 3, E3 ubiquitin protein ligase UBR1, photoreceptor outer segment membrane glycoprotein 2-like, methyltransferase-like protein 21 D, ADP-ribosylation factor 6, asparaginase homolog, kinesin family member 26 A, transmembrane protein 179, polypeptide N-acetylgalactosaminyltransferase like, CU302376.1, exonuclease 3'-5' domain containing 2, and Numb homolog protein (Figure 5).

After gene annotation, we attempted to identify conserved syntenies in the assembled region with the genomes of four fish model species, medaka, stickleback, *Tetraodon *and zebrafish. The order of the catfish genes was oriented by anchoring the scaffolds to the physical map by using BAC end sequences. One syntenic block containing seven genes (designated as J-K-L-M-N-I-H in Figure 5) and a second syntenic block containing four genes (designated as O-P-Q-R in Figure 5) are highly conserved, across most of these fish species (Figure 5). Several other smaller syntenic blocks were also somewhat conserved that contained 2-4 genes (Figure 5). However, the distance spanning these genes is different. All these 18 genes were found in catfish physical map contig of approximately 1 Mb, but these genes were found to be located in a genomic region of approximately 7 Mb in stickleback, 8 Mb in *Tetraodon*, over 24 Mb in medaka, and 44 Mb in zebrafish. Nonetheless, gene arrangements in the catfish genome are most similar to that in zebrafish, consistent with their phylogenetic relations [37].

## Conclusions

The results of this study demonstrated that for the catfish genome, the hybrid sequencing strategy using both 454 and Illumina is more effective than either alone, especially when sequencing costs were also considered, as demonstrated with the potato genome project [38]. Initially, the effectiveness of sequence assembly was almost linearly correlated with the depth of sequence coverage, but additional sequencing after a certain level (18 × 454 and 70 X Illumina) provided no additional power for effective sequence assembly. The best assembly software for 454 reads appears to be Newbler as assembly statistics resulted in the lowest number of contigs and highest values for the contig size as well as N50 value. Using the same set of assembly criteria, both ABySS and Velvet appear to be suitable for the assembly of Illumina reads. A two-step strategy, initially using Newbler for 454 reads and Velvet for Illumina reads followed by using MIRA, seemed to provide highly effective sequence assembly. Sequencing of this genomic region allowed identification of 18 protein encoding genes. Their genomic arrangements are highly conserved among catfish, zebrafish, medaka, *Tetraodon*, and stickleback.

It should be noted that this work dealt with only one million base pair region of the catfish genome. Therefore, while this work provide framework for planning of whole genome sequencing of the catfish genome, extension of technical parameters from this work to whole genome sequencing requires additional work. The combination of 454 and Illumina sequencing may not be effective in dealing with fish whose genomes are polyploidy or contain complex and long repeat structures. Even for the catfish genome, sequence assembly was attenuated with repeats, and therefore, an appropriate level of long reads, e.g., those produced by PacBio sequencing [35,39], to pass through the repeat regions may prove to be very useful.

## Methods

### DNA preparation and sequencing

Twenty four clones ensuring the coverage of a minimum tiling path of the contig0241 from the CHORI-212 BAC library [10] were selected for sequencing. The BAC DNA isolation was conducted as previously described [3], with modifications. Briefly, BAC clones were transferred from 384-well plates to a 96-well culture block, which contained 1.5 ml of 2X YT medium with 12.5 μg/ml chloramphenicol and grown at 37°C overnight with shaking at 300 rpm. The block was centrifuged at 2000 × *g *for 10 min in an Eppendorf 5804R bench top centrifuge to collect bacteria. The culture supernatant was decanted and the block was inverted and tapped gently on paper towels to remove remaining liquid. BAC DNA was isolated using the Perfectprep™ BAC 96 kit (Eppendorf North America, Westbury, NY) according to the manufacturer's specifications. An equal amount of 1 μg DNA per BAC clone was pooled, followed by purification with phenol/chloroform. This DNA was used for 454 and Illumina sequencing in the Genomic Services Lab at HudsonAlpha Institute for Biotechnology (Huntsville, AL).

### Assembly strategy

All raw 454 reads and Illumina reads were trimmed of BAC vector sequences and low quality reads were filtered before assembly. CLC Genomics Workbench was used to trim raw sequences with quality score limit of 0.01 (Q20). Illumina 72-bp paired-end reads and 454 reads shorter than 15 bp were discarded. Assembly of 454 reads alone was performed by Newbler v. 2.6, Velvet v. 1.0.01, MIRA v. 3.0.0 and CLC Genomics Work bench v. 4.0.0 (CLC Bio, Cambridge, MA). Newbler with seed length from 10 to 16, and Velvet with K-value from 25 to 95 were tested. For Illumina data, assembly of Illumina reads alone was performed by Velvet, ABySSv.1.2.1 and CLC Genomics Workbench. Velvet with K-value from 21 to 61, and ABySS with K-value from 30 to 64 were tested. The hybrid assembly with both 454 and Illumina data included three major steps: First, 454 reads alone was pre-assembled by Newbler with seed length 16, minimum overlap length 40 and minimum overlap identity 95%; second, Illumina reads were pre-assembled alone using Velvet with K-value 29. Although Velvet is able to generate scaffolded contigs, this function was turned off in this step to prevent influence of scaffolded contigs on the subsequent hybrid assembly step; third, pre-assembled 454 and Illumina contigs along with singeltons were assembled using MIRA, with minimum overlap 40.

### Comparison of assembly of paired-end reads and single-end reads

In order to compare the effects of both paired-end and single-end reads on *de novo *assembly, the Illumina paired-end sequencing data set was assembled using ABySS with a K-value 60. The same paired-end reads data set was treated as single-end reads for the comparison, by neglecting the paired-end information.

### Gene prediction and comparative analysis

Repetitive elements were masked using RepeatMasker against zebrafish repeat database. Repeat masked sequences were used for gene and syntenic identification by using both gene prediction algorithms and sequence similarity searches. GENSCAN gene model prediction algorithm was used to predict introns and exons. The resulting predictions were searched against NCBI *nr *database by using BLASTX with an E-value cutoff of 1e-10. The identified gene sequences were used for TBLASTX search against medaka, stickleback, *Tetraodon *and zebrafish peptide database with an E-value cutoff of 1e-10. The chromosomal positions of the homologous genes were identified from Ensembl database.

## Authors' contributions

YJ conducted the major part of the project including preparation of samples for sequencing, sequence analysis, assembly, and manuscript preparation. JL shared data analysis duties with YJ. EP assisted in data analysis. HK modified the manuscript. SL, SW and FS assisted in data analysis. ZL supervised the project and finalized manuscript. All authors have read and approved the final manuscript.
